# The Effects of Feed Color on Broiler Performance between Day 1 and 21

**DOI:** 10.3390/ani11061511

**Published:** 2021-05-23

**Authors:** Joseph P. Gulizia, Kevin M. Downs

**Affiliations:** School of Agriculture, Middle Tennessee State University, Murfreesboro, TN 37132, USA; jpg3y@mtmail.mtsu.edu

**Keywords:** broiler, nutrition, growth, performance, feed color

## Abstract

**Simple Summary:**

It is well established that birds, including chickens, can see in a broad range of the color spectrum, and some colors can be stimulatory. The objective of this research was to determine whether coloring feed could stimulate broiler (meat-type) chickens to consume more and, thus, grow at a greater rate. In two trials, feed was colored red, green, blue, yellow, orange, and purple. The most effective colors for increasing broiler performance were blue and purple. Other colors had little influence. Based on the results of this study, coloring feed to increase how much a broiler eats seems mostly ineffective. There may, however, be some value to further research exploring blue and purple coloring of broiler feeds.

**Abstract:**

Two trials were conducted to determine feed color effects on broiler performance. A completely randomized design was used. Trial 1 included four treatments: control (complete broiler starter diet), red, green, and blue; and Trial 2 included four treatments: control, orange, yellow, and purple. Each trial had 4 treatments with 4 replicates (60 birds/treatment) fed to 240 male Cobb 500 broilers during a 21 d grow out. Data were analyzed using the GLM procedure. In Trial 1, there were no treatment effects on average body weight, body weight gain, and feed consumption (*p* > 0.05). Adjusted feed conversion for control (1.23) was less than red (1.27; *p* = 0.001) and green (1.26; *p* = 0.009), with blue (1.25; *p* = 0.056) tending to be different during the experimental period. In Trial 2, there were no treatment effects on average body weight, feed consumption, and adjusted feed conversion during this study (*p* > 0.05). Body weight gain between d 1 to 14 for purple (490.78 g/bird) was more than orange (467 g/bird; *p* = 0.013) and yellow (461 g/bird; *p*= 0.004), with control (474 g/bird; *p* = 0.052) tending to be different. Results indicate that these feed colors had some, albeit limited, influence on broiler performance parameters.

## 1. Introduction

The nidifugous and precocial nature of chickens has equipped them with heightened senses that aid in foraging and survival. Chickens have well-developed trichromatic vision, allowing them to see all sections of the visible light spectrum and some ultraviolet [1,2,3,4,5,6,7,8]. In addition to color discrimination, poultry memorize certain color traits [9,10]. Güntürkün [11] explored the physiology of avian eyesight and showed that incoming light will pass through four optic structures of the eye: the cornea, anterior chamber, lens, and vitreous body. These structures are perceived to transmit near ultraviolet (UV) range wavelengths at approximately 310 nm [12]. Avian species experience increased visual occurrences with shortwave reflectance [13,14]. Specialized cone cells afford birds the ability to detect these shorter wavelengths. Birds having the ability to visually differentiate colors is implied by the presence of four spectrally different cones (red, green, blue, and ultraviolet) with unique visual pigments and oil droplets that provide spectral sensitivity ranging from 350 to 780 nm [15,16,17,18]. These oil droplets contain carotenoid pigments that can screen extraneous wavelengths and prevent damage from UV light entering the bird’s eye. It is beyond the scope of this article to address in-depth aspects of avian eye physiology. This topic has been extensively reviewed and reader is referred to Güntürkün [11] and Martin and Osorio [19]. 

Investigations by Ham and Osorio [20] have revealed that simple tasks by birds (e.g., pecking) can be influenced by color and can be utilized for increased interest in a particular food item. The well-developed eyesight of avian species indicates the potential to utilize colors as a stimulant to increase a bird’s response. Goldsmith [16] demonstrated when budgerigars (*Melopsittacus undulates*) were trained to associate a feed reward with a yellow light, the birds readily selected the correct light among a mixture of red and green lights in a random sequence. However, when the mixture of lights contained a higher percentage of red, the birds became erratic with their choices. They repeated the study using a violet light as the indicator for a food reward. The budgerigars were observed to successfully select the violet light at higher color mixtures as compared to yellow light. Birds are perceived to visually see in near ultraviolet wavelengths and have the visual system capacity to envision colors that humans cannot [16]. This heightened sense of sight allows birds to visually distinguish their environment and memorize color characteristics, potentially leading to color-based preferences [17,21]. Utilizing color as a natural stimulant in the form of colored feeds has the potential to increase commercial poultry performance through increased feed consumption [21]. Leslie et al. [22] concluded that utilizing feed color can potentially alter feed consumption during different feeding stages. Most previous research regarding feed color effects on performance of broiler chickens is older and limited. 

Khosravinia [5] observed that broilers consumed significantly more feed with green lighting and green feed over other light and feed combinations. Additionally, Cooper [23], in an older study, explored feed colors and neophobia in turkeys. The author reported that turkeys preferred green colors, as indicated by their strong acceptance of green feed. White Leghorns were tested on their response to red, yellow, green, and blue feed colors [21]. The birds preferred blue feed the most and red the least, with red feed significantly decreasing feed consumption. In contrast, Leslie et al. [22] found that when broiler chicks were given a choice between a non-colored and colored feed, they preferred non-colored diets. Although somewhat inconsistent, previous research has demonstrated that feed color has the potential to increase or decrease feed consumption in broilers, thus affecting body weight gain during a growing period [5,21,23]. In an effort to expand the base of knowledge related to coloration of poultry feeds, this research was undertaken to assess how altered feed color may impact the performance of broilers grown to 21 days of age. Color induced increases in broiler feed consumption, with a concomitant performance improvement, could prove a useful tool in modern poultry production. Colors were selected for this research to represent primary and secondary colors spanning a wide swath of the visible color spectrum and representing longer (red, orange, yellow, green) and shorter wavelengths (blue and purple).

## 2. Materials and Methods

### 2.1. Animal Welfare and Handling

Study design and animal handling procedures were approved by the Middle Tennessee State University Institutional Animal Care and Use Committee (IACUC: PRN 21–2002) and conformed to accepted practices [24,25]. Birds were euthanized by cervical dislocation in accordance with American Veterinary Medical Association Guidelines [26].

### 2.2. Diet Preparation

In this research, 2 separate trials were conducted with the only difference being feed colors. Dietary treatments for Trial 1 included control (basal diet), red, green, and blue. Dietary treatments for Trial 2 included control (basal diet), orange, yellow, and purple. Dietary color treatments consisted of added non-nutritive human food-grade powdered dyes (LorAnn Oils^®^, Lansing, MI, USA). Control diets had a 0% color dye inclusion. Color specifications are shown in Table 1. 

Each dye color was mixed into the basal diet and dispersed using a tumble mixer on the crumbled feed to ensure a uniform color. The basal diet was a proprietary typical U.S. broiler industry standard complete commercial broiler starter diet (corn-soybean meal based; crumbled) obtained from a local broiler complex feed mill. The feed used in both trials met or exceeded National Research Council requirements [27] and standard nutrient recommendations for the broiler strain used in this research (Table 2). The basal diet used in this study (both trials) was single-batch sourced. Diets used in both experiments were isocaloric and isonitrogenous.

### 2.3. Bird Housing and Performance Determination 

For Trials 1 and 2, 240 male Cobb 500 broilers (per trial) obtained from a local Cobb hatchery were grown for 21 d in standard wire-floor battery pens. Each growing pen (0.79 m^2^) contained 15 broilers (526.67 cm^2^/bird), with birds being randomly assigned to each. A treatment was randomly assigned to each battery cage and replicated 4 times, totaling 16 experimental units. Birds were brooded at approximately 35 °C, with temperature reduced approximately 3 °C each week. Room temperature was kept at 27 °C. Continuous white light (24 L:0 D; 25 lux) was provided and water offered ad libitum. Color of room walls were white and feed troughs gray. Broilers were fed ad libitum for each treatment from d 1 to 8. Feed access was limited to 22 h on d 9, 18h on d 10, 16h from d 11 to 18, and increased to 20 h per day from d 19 to 21. Feeder space was 9.6 cm/bird throughout the growing period. 

Birds and feed were weighed on d 1, 7, 14, and 21 to determine body weight gain (BWG), feed consumption, and feed conversion ratio (FCR). Feed consumption was calculated as the difference between feed offered and residual feed. Cumulative feed consumption (CFC) was determined by calculating the difference between feed offered and feed which remained between d 1 to 7, 1 to 14, and 1 to 21. Feed conversion ratio was calculated using feed consumption and BWG and was adjusted for mortality (AFCR).

### 2.4. Statistical Analysis

Data were analyzed as a completely randomized design with battery pen representing the experimental unit. Treatment main effect significance was determined using the GLM procedure of the SAS statistical package [28]. Mortality was arcsine transformed before analysis. Treatment means were further separated using Tukey’s HSD test. Significance level was set at *p* ≤ 0.05. Tendencies were set at 0.05 < *p* < 0.10. All data were analyzed for normality using the Shapiro–Wilk test as a component of the univariate analysis and results obtained were presented as least squares means with their pooled standard errors of mean (SEM).

## 3. Results

For Trial 1, little effects of feed color on bird performance were recorded (Table 3). Most observed differences appeared to reside with feed color effects on feed conversion. Based on the results of this study, red and green feed depressed d 1 to 21 AFCR by 3.2 and 2.4% (*p* < 0.05), respectively, compared to the control diet. A similar effect from red feed was seen for AFCR between d 1 and 14, with a 2.6% increase compared with control (*p* < 0.05). Interestingly, birds consuming the blue colored feed had similar AFCR to control birds at every time period. However, no other performance parameters differed across treatments in this trial. Dietary color treatments had no influence on bird mortality throughout the 21 day growing period (*p* > 0.05).

Similar to the results of Trial 1, Trial 2 showed minimal influences of feed colored orange, yellow, or purple on overall bird performance compared to the control diet (Table 4). However, some interesting effects were detected. The BWG between d 1 and 14 of birds consuming the purple colored feed was 6.4% higher than birds consuming yellow (*p* < 0.05). A similar trend was observed for d 7 to 14 BWG (*p* < 0.05). Much like Trial 1, feed color dietary treatments had no influence on bird mortality throughout this study (*p* > 0.05).

## 4. Discussion

Much of the previous research to assess feed color influences in poultry has focused on the layer or the turkey, with limited broiler data. Further, much of the feed color research in broilers is not current and lacks a focus on the uniqueness of the modern broiler strains. With that said, there are both some consistencies and inconsistencies between the results of the present study and previous work. Much of that is likely due to variation in growing period length and broiler strain.

Toghyani and Mesmarian [29] utilized broilers to assess the effects of red, green, and blue feed colors on growth performance variables. The authors reported increased feed consumption in broilers fed a colored diet, leading to the colored feed groups having an overall heavier body weight than control birds. Likewise, previous research reports that feed color effect on ABW is a result of the effects on CFC [23,29]. Results from the present study are consistent for ABW, but with an observed lack of significance for CFC.

There were no feed color effects observed for ABW during either Trial 1 or 2 during the entire experimental period. This present research does not provide data to support coloring of feed to increase ABW. However, previous research demonstrates that feed color effects have the potential to alter CFC while subsequently affecting ABW.

For Trial 1 and 2, there were no observed feed color effects for CFC of broilers fed colored diets. Results from this study indicate that CFC of modern strain broilers were not influenced by color and color intensities utilized in this research. However, researchers have reported feed consumption effects for poultry when using colored feed as a natural stimulant. Hurnik et al. [21] observed feed color as a stimulus for feed consumption in White Leghorn pullets. Birds were observed to prefer blue > green > yellow > red feed, with the highest feed consumption occurring with a blue colored diet and lowest on red. Furthermore, Weeks et al. [30] observed a color effect on feed consumption when feeding layer and broiler chicks red, blue, green, and yellow colored diets. Their study showed birds consumed significantly more yellow colored feed and less blue. They concluded broilers preferred yellow feed and layers preferred blue feed throughout the study. The increase in consumption of yellow feed was inferred as an effect of natural colored grains birds may inherently experience when foraging, and blue feed as birds are more influenced by shortwave UV reflectance [13,14,30]. In the present study, broilers did not have the ability to preferentially select feed based on color, which can be important in isolating the inherent stimulatory color effects birds may experience. Avian species use visual cues like color to select beneficial and avoid potentially harmful food [31,32]. Broiler performance results from this study suggest feed color effects are inconsistent, but do not indicate an aversion to certain colors. Leslie et al. [22] fed a colored and non-colored grower diet to male broilers, preventing birds from having a preference during the grower phase. The authors reported no consistent feed color effects on feed consumption. However, they observed a single multicolored diet influenced feed consumption, but this effect did not influence BWG. The lack of significance in the present research observed for feed color effects on CFC could be influenced by feed color intensity and lighting color used in this study. The intensities of colors perceived by birds may not have been high enough to stimulate an effect on CFC. Likewise, a more profound effect on CFC may have occurred if feed color was combined with varying lighting colors. Although the present study resulted in no effects on CFC, blue and purple feed colors used in these trials were observed to minimally affect BWG and AFCR. 

A paucity of previous research has demonstrated effects on BWG through the manipulation of feed color. Cooper [23] reported that turkey poults fed a green colored feed over a non-colored control diet resulted in birds having a significantly higher BWG. Additionally, the effects on BWG for birds fed purple could be a result of their increased shortwave reflectance vision [13,14]. This attribute allows visual differentiation of UV and blue better than the rest of the color spectrum, which may have influenced broiler performance in this study.

Toghyani and Mesmarian [29] reported that broiler chicks fed a control and blue colored diet resulted in the lowest FCR when compared to birds fed a red or green colored feed during a 42 d grow out. Results from the present study and previous research indicate that blue colored feed has favorable effects on AFCR for modern broiler strains [29]. This observed blue feed color effect is attributed to birds being more influenced by UV and blue colors as compared to the remaining color spectrum [13,14]. 

Potential mortality effects from feed coloring were not observed during either trial. This is consistent with previous research using an additive to color feed [17,21,30,32]. Cooper [23], however, observed that turkey poults consuming a green-dyed feed had a 2.5% greater mortality than those fed a non-dyed feed, but they did not correlate the effects of green feed with increased bird mortality. Researchers throughout the years have used different methods to alter feed color effectively and safely for bird consumption.

There is a paucity of research on feed color influences on broiler performance, with most focused on layers and turkeys. Further, little data exist on feed color and its effect on the modern broiler strain. Previous researchers have mainly assessed feed color effects on broilers grown for more than 35 d [22,29,30]. The present work specifically assessed feed color influences during the early growth phase, which is likely reflected in the results. Broiler age may interact with feed color and researchers envisage future work focused on feed color effects during a longer growing period, with different feed forms, and various feeding regimens.

## 5. Conclusions

Overall, this research showed inconsistent feed color effects on broiler performance parameters. Blue and purple feed colors were found at some level to positively influence feed conversion and body weight gain more than other feed colors. However, a majority of the broiler performance parameters were not influenced by feed color. Blue and purple feed colors appear to hold the most promise for potential broiler performance effects, but this needs to be further fleshed out. Additionally, the practicality of coloring feeds at the commercial feed mill level may pose some challenges to broiler complexes.

## Figures and Tables

**Table 1:** Color Specifications for Each Diet Treated with a Non-Nutritive Food-Grade Dye (Trials 1 and 2).

Color	Hexadecimal Color Code	RGB Color Model
Red	#bb5a58	73.33% red
		35.29% green
		34.51% blue
Green	#739a2d	45.10% red
		60.39% green
		17.65% blue
Blue	#4a9c9d	29.02% red
		61.18% green
		61.57% blue
Orange	#f59b54	96.08% red
		60.78% green
		32.94% blue
Yellow	#e5c92a	89.80% red
		78.82% green
		16.47% blue
Purple	#6b5669	41.96% red
		33.73% green
		41.18% blue

**Table 2:** Analyzed Nutrient Composition of Basal Diet (Dry Matter Basis) (Trials 1 and 2).

Analysis	Control
Dry matter (DM), %	88.4
Crude protein (CP), %	27.3
Metabolizable energy (ME), kcal/kg	3530
Neutral detergent fiber, %	9.6
Ca, %	0.89
P, %	0.79
Mg, %	0.19
K, %	1.18
Na, %	0.195
Fe, ppm	133
Zn, ppm	175
Cu, ppm	47
Mn, ppm	186
Mo, ppm	2.5

**Table 3:** Influence of Control (basal diet only), Red, Green, and Blue Feed Colors on Average Body Weight, Cumulative Feed Consumption, Body Weight Gain, and Adjusted Feed Conversion Ratio of Broilers from 1 to 21 Days of Age (Least Squares Means) (Trial 1).

Item	Control	Red	Green	Blue	P_r_ > F	SEM ^A^
ABW ^B^, g/bird						
Day 1	43.8	44.2	44	44.4	0.72	0.38
Day 7	202	201	199	205	0.606	3.3
Day 14	556	548	548	547	0.813	6.9
Day 21	1087	1055	1066	1064	0.448	14
CFC ^B^, g/bird						
Day 1 to 7	165	163	162	164	0.966	4.2
Day 1 to 14	585	581	581	571	0.796	10.2
Day 1 to 21	1270	1255	1269	1254	0.896	19.2
BWG ^B^, g/bird						
Day 1 to 7	157	157	155	159	0.695	4
Day 7 to 14	355	339	346	343	0.728	11.7
Day 1 to 14	509	494	496	498	0.779	14.8
Day 14 to 21	526	506	517	516	0.281	12.4
Day 1 to 21	1029	987	1009	1009	0.623	25.9
AFCR ^B^, g:g						
Day 1 to 7	1.05	1.04	1.05	1.03	0.738	0.012
Day 7 to 14	1.20 ^b^	1.24 ^a^	1.23 ^ab^	1.22 ^ab^	0.059	0.011
Day 1 to 14	1.15	1.18	1.17	1.15	0.058	0.008
Day 14 to 21	1.30 ^c^	1.37 ^ab^	1.34 ^ab^	1.34 ^bc^	0.011	0.011
Day 1 to 21	1.23 ^c^	1.27 ^ab^	1.26 ^ab^	1.25 ^bc^	0.006	0.007
Mortality, %						
Day 1 to 21	6.7	8.3	6.7	5	0.887	2.97

^A^ Standard error of the mean; ^B^ ABW = average body weight; CFC = cumulative feed consumption; BWG = body weight gain; AFCR = mortality adjusted feed conversion ratio; ^abc^ means in the same row with different superscript letters are different (*p* < 0.05).

**Table 4:** Influence of Control (basal diet only), Orange, Yellow, and Purple Feed Colors on Average Body Weight, Cumulative Feed Consumption, Body Weight Gain and Adjusted Feed Conversion Ratio of Broilers from 1 to 21 Days of Age (Least Squares Means) (Trial 2).

Item	Control	Orange	Yellow	Purple	P_r_ > F	SEM ^A^
ABW ^B^, g/bird						
Day 1	41.8	41.9	42	41.9	0.998	0.42
Day 7	186	185	182	192	0.457	4.1
Day 14	520	509	506	525	0.147	6.1
Day 21	998	993	998	1014	0.723	14.4
CFC ^B^, g/bird						
Day 1 to 7	156	153	149	158	0.242	2.9
Day 1 to 14	547	542	529	555	0.164	7
Day 1 to 21	1214	1202	1196	1228	0.261	11.4
BWG ^B^, g/bird						
Day 1 to 7	144	143	140	150	0.41	3.8
Day 7 to 14	338 ^a^	324 ^ab^	322 ^b^	339 ^a^	0.004	2.9
Day 1 to 14	474 ^ab^	467 ^ab^	461 ^b^	491 ^a^	0.02	5.4
Day 14 to 21	478	478	483	489	0.82	9.5
Day 1 to 21	951	944	940	968	0.329	11.7
AFCR ^B^, g:g						
Day 1 to 7	1.09	1.07	1.06	1.05	0.536	0.015
Day 7 to 14	1.18	1.2	1.18	1.2	0.629	0.013
Day 1 to 14	1.15	1.16	1.15	1.15	0.666	0.008
Day 14 to 21	1.4	1.39	1.38	1.39	0.923	0.024
Day 1 to 21	1.28	1.27	1.27	1.27	0.917	0.012
Mortality, %						
Day 1 to 21	1.7	1.3	4.4	1.7	0.607	1.68

^A^ Standard error of the mean; ^B^ ABW = average body weight; CFC = cumulative feed consumption; BWG = body weight gain; AFCR = mortality adjusted feed conversion ratio; ^a,b^ means in the same row with different superscript letters are different (*p* < 0.05).

## Data Availability

All data are contained within the article.

## References

[1] Bell D.J., Freeman B.M. (1971). Physiology and Biochemistry of the Domestic Fowl.

[2] Cornsweet T.N. (1970). Chapter 3: The physics of light. Visual Perception.

[3] Guhl A.M., Hafez E.S.E. (1962). The behaviour of chickens. The Behavior of Domestic Animals.

[4] Gunter W.C. (1964). Further observations on effect on high incubation temperature on frequency of pecking and color preference in chicks. Proc. Indiana Acad. Sci..

[5] Khosravinia H. (2007). Preference of Broiler Chicks for Color of Lighting and Feed. J. Poult. Sci..

[6] Kilham P., Klopfer P.H., Oelke H. (1968). Species identification and colour preferences in chicks. Anim. Behav..

[7] Malott M.K. (1968). Stimulus control in stimulus-deprived chickens. J. Comp. Physiol. Psychol..

[8] Taylor A., Sluckin W., Hewitt R. (1969). Changing colour preferences of chicks. Anim. Behav..

[9] Aoki M., Izawa E.-I., Koga K., Yanagihara S., Matsushima T. (2000). Accurate Visual Memory of Colors in Controlling the Pecking Behavior of Quail Chicks. Zool. Sci..

[10] Osorio D., Jones C.D., Vorobyev M. (1999). Accurate memory for colour but not pattern contrast in chicks. Curr. Biol..

[11] Güntürkün O., Whittow G.C. (2000). Sensory Physiology: Vision. Sturkie’s Avian Physiology.

[12] Emmerton J., Schwemer J., Muth I., Schlecht P. (1980). Spectral transmission of the ocular media of the pigeon (Columba livia). Invest. Ophtalmol. Visual Sci..

[13] Delhey K., Hall M., Kingma S.A., Peters A. (2013). Increased conspicuousness can explain the match between visual sensitivities and blue plumage colours in fairy-wrens. Proc. Biol. Sci..

[14] Egbuniwe I., Ayo J. (2016). Physiological roles of avian eyes in light perception and their responses to photoperiodicity. World’s Poult. Sci. J..

[15] Chen D.M., Goldsmith T.H. (1986). Four spectral classes of cone in the retinas of birds. J. Comp. Physiol. A.

[16] Goldsmith T.H. (2006). What Birds See. Sci. Am..

[17] Lecuelle S., Leterrier C., Chagneau A.M., Laviron F., Lescoat P., Bastianelli D., Bertin A., Bouvarel I. (2011). Experience with a variety of feed colours reduces feed neophobia in the turkey. Appl. Anim. Behav. Sci..

[18] Prescott N.B., Wathes C.M., Jarvis J.R. (2003). Light, vision and the welfare of poultry. Anim. Welf..

[19] Martin G.R., Osorio D., Masland R.H., Dallos P., Firestein S., Bushnell M.C., Kaas J.H. (2008). Vision in birds. The Senses: A Comprehensive Reference.

[20] Ham A., Osorio D. (2007). Colour preferences and colour vision in poultry chicks. Proc. R. Soc. B: Boil. Sci..

[21] Hurnik J.F., Jerome F.N., Reinhart B.S., Summers J.D. (1971). Color as a Stimulus for Feed Consumption. Poult. Sci..

[22] Leslie A.J., Hurnik J.F., Summers J.D. (1973). Effects of color on consumption of broiler diets containing rapeseed meal and rapeseed. Can. J. Anim. Sci..

[23] Cooper J.B. (1971). Colored Feed for Turkey Poults. Poult. Sci..

[24] American Society of Animal Science/American Dairy Science Association/Poultry Science Association (2020). Chapter 3: Husbandry, Housing, and Biosecurity. Guide for the Care and Use of Agricultural Animals in Agricultural Research and Teaching.

[25] American Society of Animal Science/American Dairy Science Association/Poultry Science Association (2020). Chapter 4: Environmental Enrichment. Guide for the Care and Use of Agricultural Animals in Agricultural Research and Teaching.

[26] American Veterinary Medical Association (2020). AVMA Guidelines for the Euthanasia of Animals.

[27] National Research Council (1994). Nutrient Requirements of Poultry.

[28] SAS Institute (2017). SAS/STAT® User’s Guide Version 14.3.

[29] Toghyani M., Mesmarian M.A. Effect of feed color on growth performance and tonic immobility in broiler chicks. Proceedings of the XVth European Poultry Conference.

[30] Weeks C.A., Brooks C., Coe G., Danbury T.C. (1997). Effects of food and feeder colour on food consumption of young layers and broilers. Br. Poult. Sci..

[31] Gentle M.J. (1985). Sensory involvement in the control of food intake in poultry. Proc. Nutr. Soc..

[32] Roper T.J., Marples N.M. (1997). Colour preferences of domestic chicks in relation to food and water presentation. Appl. Anim. Behav. Sci..

